# Maternal depression and anxiety predicts the pattern of offspring symptoms during their transition to adulthood

**DOI:** 10.1017/S0033291715001956

**Published:** 2015-10-12

**Authors:** H. Gonçalves, R. M. Pearson, B. L. Horta, D. A. González-Chica, E. Castilho, M. Damiani, R. C. Lima, D. P. Gigante, F. C. Barros, A. Stein, C. G. Victora

**Affiliations:** 1 Postgraduate Program in Epidemiology, Universidade Federal de Pelotas, Pelotas, Rio Grande do Sul, Brazil; 2 School of Social and Community Medicine, Bristol University, UK; 3 Postgraduate Program in Nutrition, Universidade Federal de Santa Catarina, Brasil; 4 Postgraduate Program in Education, Universidade Federal de Pelotas, Brasil; 5 Postgraduate Program in Health Science, Universidade Federal da Grande Dourados, Mato Grosso do Sul, Brasil; 6 Postgraduate Course in Health and Behavior, Universidade Católica de Pelotas, Pelotas, Brazil; 7 Depertment of Psychiatry, Oxford Univeristy, UK

**Keywords:** Cohort study, common mental disorder, intergenerational, mental health, offspring

## Abstract

**Background:**

Episodes of depression and anxiety (D&A) during the transition from late adolescence to adulthood, particularly when persistent, are predictive of long-term disorders and associated public health burden. Understanding risk factors at this time is important to guide intervention. The current objective was to investigate the associations between maternal symptoms of D&A with offspring symptoms during their transition to adulthood.

**Method:**

Data from a large population-based birth cohort study, in South Brazil, were used. Prospective associations between maternal D&A and offspring risk of these symptoms during the transition to adulthood (18/19, 24 and 30 years) were estimated.

**Results:**

Maternal D&A in adolescence was associated with offspring symptoms across the transition to adulthood, associations were consistently stronger for females than for males. Daughters whose mothers reported D&A were 4.6 times (95% confidence interval 2.71–7.84) as likely to report D&A at all three time-points, than daughters of symptom-free mothers.

**Conclusions:**

Maternal D&A is associated with persistent D&A during the daughter's transition to adulthood. Intervention strategies should consider the mother's mental health.

## Introduction

Depression and anxiety (D&A) is responsible for a substantial proportion of the global burden of mortality and disability (WHO, 2001, 2010). Presentation of symptoms during late adolescence is associated with an increased risk of these disorders throughout adulthood and adults often experience their first episode in adolescence. The transition to adulthood is therefore one important period where the burden of these disorders emerges (Patton *et al.* 2014). Interventions at this time could reduce both the short- and long-term impairment. Despite this importance, there is little detailed knowledge from prospective studies about risk factors for D&A across the transition to adulthood (Thapar *et al.* 2012; Patton *et al.* 2014).

Previous evidence has highlighted that the persistence of episodes of D&A during the transition to adulthood is particularly predictive of later risk, with isolated episodes in adolescence more often resolving naturally (Patton *et al.* 2014). Identifying modifiable risk factors for the persistence of these disorders, is crucial to highlight opportunities for interventions during this important time (Patton *et al.* 2014).

Before adolescence there are no gender differences in the incidence of D&A. In contrast, by late adolescence the risk in females is at least double that for males; a risk ratio which persists into adulthood (Thapar *et al.* 2012). The transition to adulthood is thus a particularly vulnerable time for females. Identifying gender-specific risk factors at this time is important to reduce the risk in females. This is particularly important in middle-income countries where the female vulnerability adulthood appears greatest (Rojas *et al.* 2005).

One potentially modifiable risk factor is maternal mental health. There is substantial evidence that maternal D&A increases the risk of mental disorders among the offspring (Goodman & Gotlib, 1999; Isohanni *et al.* 2000; Beardslee *et al.* 2003; Ramchandani & Stein, 2003; Elgar *et al.* 2004*a*, *b*; Weissman *et al.* 2006; Goodman, 2007; Goodman *et al*. 2011; Schepman *et al.* 2011; Pearson *et al.* 2013), However, the role of maternal mental health during the child's transition into adulthood has not been examined in detail. To our knowledge there is only one previous prospective study to investigate this issue, the 1970 British birth cohort (Schepman *et al.* 2011). In this study, maternal emotional problems, measured when the offspring were aged 16 and 30 years, were correlated with concurrent adolescent emotional problems. Maternal scores at offspring age 16 years were also associated with later offspring scores at age 30 years (Schepman *et al.* 2011). However, despite previous evidence that associations may be stronger in daughters, in this study there were no gender differences.

The present study aimed to build on these previous findings and compare maternal and offspring associations in the context of a large population-based birth cohort study in a middle-income setting. This is important because we cannot assume that associations found in high-income countries are the same in low- and middle-income countries. The current report provides the first data from a large middle-income cohort. Investigations of the same associations widely reported in UK and US studies in a different context improve our understanding. Cross-cohort comparisons can provide important information regarding the role of different cultural and socio-economic environments.

In addition the study aimed to provide a more detailed analysis of the patterns of the associations in three ways. First, we investigated the impact of maternal depression in both childhood and adolescence on offspring symptoms. Second, we investigated the association between maternal symptoms and the number of offspring episodes of D&A as well as comparing the trajectories of offspring symptom scores from ages 19 to 30. This is important given the evidence that persisting symptoms are particularly associated with future risk (Patton *et al.* 2014). Finally, in a more explorative analysis, we investigated whether any particular constellation of maternal symptoms account for more of the variance in any observed inter-generational associations, than other symptom constellations. For example, there is previous evidence that depressed cognitions mediate associations between maternal and offspring depression (Pearson *et al.* 2013). This could provide further hypotheses which in the future may help inform more focused intervention targets.

## Method

### Sample

Pelotas is a southern Brazilian city with a population of 3 28 000 inhabitants. In 1982, all maternity hospitals located in the city were visited daily and live-born children whose families lived in the urban area of the city were examined and their mothers interviewed. Over 99% of all births took place in these hospitals, and among those contacted the refusal rate to take part in the 1982 Cohort was lower than 1%. The starting sample of the 1982 Cohort was 5914 mothers. This cohort has been followed up on several occasions (Victora & Barros, 2006). The Ethical Review Board of the Faculty of Medicine of the Federal University of Pelotas approved the study, and written informed consent was obtained from participating subjects. In 1982, verbal consent was obtained from the mothers, as this was the standard practice at that time.

In 1986, a city-wide census was carried out in search of children belonging to the cohort and 84% of the original cohort members were located. A systematic sample of 403 mothers contacted on that occasion were invited to participate in a sub-study on mental health and child development (Damiani, 1988) and 347 mothers answered the Self-Reporting Questionnaire (SRQ)-20 when the mean age of their children was 3.6 years. In 2000, male subjects answered the SRQ-20 test when they undertook the compulsory Army entrance examination. In 2001, all households belonging to a systematic sample of 27% of urban census tracts were visited. Male and female cohort members were identified and their mothers answered the SRQ-20 questionnaire (Mari & Williams, 1986). Female cohort members living in these tracts also answered the questionnaire. In 2003–2004, using information from existing addresses and also relying on an intense mass media campaign, all cohort members were invited to visit a research clinic for a comprehensive assessment, which included the SRQ-20. The follow-up rate on this occasion was approximately 75%. In 2012, cohort members were invited to visit a clinic for a comprehensive examination; the follow up rate was 68%.

As can be seen in Table 1, there was no evidence that the samples with maternal complete data at 4 years *or* 18/19 years differed from each other or from the rest of the cohort, with respect to socio-demographic characteristics. Most importantly, there was also no evidence that offspring SRQ-20 scores were different across these samples. Therefore, there is no reason to believe that these samples are biased with respect to associations with offspring D&A.

**Table 1.** Comparison of samples with and without maternal SRQ-20 data

	(1) Sample with maternal SRQ-20 at age 4 (*n* = 347)	(2) Sample with maternal SRQ-20 at age 18/19 but not age 4 (*n* = 1453)	(3) Sample without maternal SRQ-20 at either time-points (*n* = 4114)	Inferential statistics
Offspring SRQ-20 at age 24, mean (s.d.)	4.6 (3.8)	4.8 (4.1)	4.7 (3.9)	Sample 1 compared to sample 3, *p* = 0.874
				Sample 2 compared to sample 3, *p* = 0.371
Household asset index – quintiles				
1	18%	19%	21%	
2	22%	19%	20%	
3	18%	22%	19%	
4	21%	22%	19%	
5	21%	19%	21%	*χ*^2^ = 13.6, *p* = 0.093
Family income in minimum wages				
⩽1	21%	20%	23%	
1.1–3	48%	49%	47%	
3.1–6	20%	20%	18%	
6.1–10	6%	6%	6%	
>10	4%	5%	6%	*χ*^2^ *p* = 0.129

### Measures

#### The SRQ-20

**Fig. 1.**
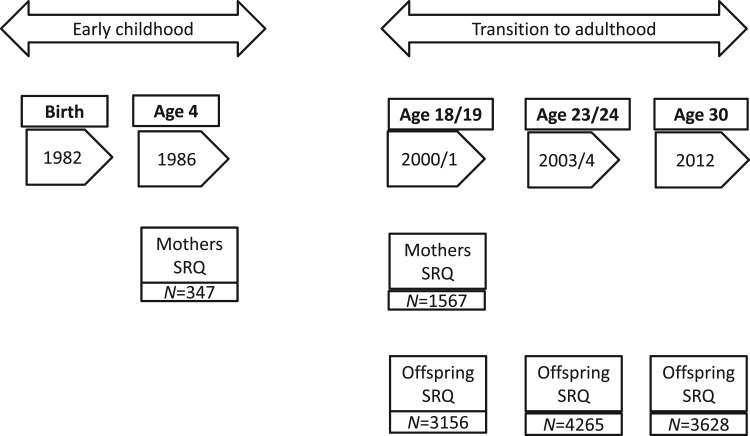
Timing of SRQ-20 measurements in mothers and offspring.

The SRQ-20 was used to assess the presence of D&A in cohort members and their mothers (Mari & Williams, 1986; Patel *et al.* 1999). The SRQ-20 test consists of 20 questions about physical and psychological symptoms during the 30 days prior to the interview (Mari & Williams, 1986). Males who presented ⩾6 and females who had ⩾8 positive answers were considered as indicative of a possible D&A disorder (Patel *et al.* 1999). These thresholds for the SRQ-20 have good sensitivity (83%) and specificity (80%) for indicating disorder against a psychiatric interview (Mari & Williams, 1986). These validation studies demonstrated that a lower threshold for males is required given that males tend to underreport symptoms on screening tools. Symptom constellation scores were also derived using the sum of specific items relating to the following core mental health symptoms based on ICD-10 criteria: depressed thoughts/cognitions; low mood/anhedonia; somatic symptoms; anxiety; sleep problems/fatigue and poor concentration. Subjects were categorized as experiencing the symptom if they endorsed ⩾2 symptom-specific items. The SRQ-20 was completed by the cohort mothers when offspring were approximately 4 and 18/19 years old and by their offspring at age 18/19, 23/24 and again at age 30. More details of the sample sizes, recruitment and assessment of mothers and offspring at each of these time-points is given in Fig. 1.

#### Confounding variables

Information on confounding variables was collected in the early phases of the study: family income at birth in multiples of 1982 minimum wage (with one minimum wage being equivalent to US$50 a month in 1982), maternal schooling (information on the highest grade of schooling successfully completed) and household assets index (obtained through factor analysis and based on the ownership of household goods). Such measures were also repeated in 2004 (cohort member age 22) and to account for potential changes in circumstances we included both timings.

### Analysis

Our first question relates to risk of D&A disorder among the offspring at the three different ages, according to maternal disorder when the child was (1) age 4 and (2) age 18/19. For these main effects we use a binary outcome: D&A or not. As D&A is a common outcome, Poisson regression is used as this estimates prevalence ratios (PRs) rather than odds ratios which provide a more accurate representation of relative risk with common outcomes. This analysis estimates the extent to which maternal depression increases risk of D&A at each individual age. We were also interested in the role of offspring gender. To formally test for the effect of modification × offspring gender, maternal depression × offspring gender, interaction terms were included in regression models. The analyses were adjusted for confounding by family income, maternal schooling and household wealth index. In addition, correlation coefficients between continuous SRQ-20 scores of mothers and offspring were also estimated separately for each gender.

Where statistical evidence for associations was present, three further set of analyses were conducted. First, the PRs of disorder in offspring according to specific symptom constellations in the mother in late adolescence, were estimated using Poisson regression models as above. Each of the maternal symptom constellations was included in the same model, so that estimates represent ‘independent’ associations. Second, persistence of offspring D&A across the three time-points were investigated by deriving a variable comprising the number of episodes (0, 1, 2 or 3) the subject experienced. Ordinal and multinomial and logistic regression models were used to investigate the association between maternal disorder and persistence of offspring D&A. For this question of chronicity we look at the number of episodes of D&A as an outcome, we therefore use ordinal logistic regression as the outcome has more than two categories which are ordered. Multinomial regression provides further information regarding the risk at each level of the ordinal variable (i.e. each number of episodes, 1, 2 or 3).

Finally, we use linear random-effects regression models [xtmixed command in Stata v. 13 (StataCorp., USA)] to model the pattern of continuous symptom scores within subjects (intercept and slope of SRQ-20 trajectories from age 18/19 to 30) and whether these parameters vary according to between subject factors: maternal D&A. These models incorporate the repeated measure nature of these data allowing us to look at all timings together and how maternal depression influences the change in symptoms over time.

The different analytical approaches reflect different types of data needed to answer questions relating to different aspects of offspring mental health. We look at the impact of maternal depression for offspring risks at three individual timings, then the number of offspring episodes and the finally trajectories of symptoms across the repeated measures.

## Ethical standards

The authors assert that all procedures contributing to this work comply with the ethical standards of the relevant national and institutional committees on human experimentation and with the Helsinki Declaration of 1975, as revised in 2008.

## Results

### Descriptive results

Table 2 shows that the prevalence of maternal D&A was higher among mothers with low family income and schooling.

**Table 2.** Prevalence of maternal depression and anxiety according to maternal and socioeconomic variables in 1982

Socioeconomic variables	Prevalence of maternal depression and anxiety	
	1986	2000–01
Family income in minimum wages	*p* = 0.02**	*p* < 0.001*
⩽1	33.8%	44.9%
1.1–3	32.1%	33.8%
3.1–6	27.1%	32.0%
6.1–10	4.6%	24.4%
>10	6.7%	11.9%
Maternal schooling in years	*p* < 0.001*	*p* < 0.001*
0–4	41.4%	43.4%
5–8	30.6%	35.9%
⩽9	10.3%	18.3%
Household asset index – quintiles	*p* = 0.02**	*p* < 0.001*
1st	38.2%	62.5%
2nd	45.2%	50.0%
3rd	22.2%	34.3%
4th	13.2%	33.5%
5th	22.7%	28.3%

* Test for linear trend, ** test for heterogeneity.

### Maternal D&A and offspring D&A from ages 18/19 to 30

Taking the later SRQ-20 measure at age 30 as the outcome measure, there was no evidence for an interaction between gender × maternal disorder in early childhood (interaction term after adjustments = 1.19, *p* = 0.736). However, after adjustments there was statistical evidence for a gender × maternal disorder in adolescence interaction (interaction term 1.77, *p* = 0.026), with a stronger association for females. A similar pattern was found when looking at alternative timings of offspring symptoms.

For female offspring, there was clear statistical evidence that maternal disorder presenting in adolescence was associated with increased prevalence of D&A at 19, 23–24 years and 30 years. A similar pattern was seen for associations with maternal disorders reported in childhood; however, possibly due to the smaller sample size, these associations were not significant (see Table 3).

**Table 3.** Prevalence ratio (PR) of depression/anxiety among male and female subjects according to maternal depression/anxiety

Maternal depression/anxiety	18 years (2000)			23–24 years (2004–2005)			30 years (2012)		
	*N*	Crude PR	Adjusted PR^a^	*N*	Crude PR	Adjusted PR^a^	*N*	Crude PR	Adjusted PR^a^
Males									
1986 (aged 4)		*p* = 0.05	*p* = 0.21		*p* = 0.06	*p* = 0.13		*p* = 0.630	*p* = 0.923
No	107	1.00	1.00	99	1.00	1.00	80	1.00	1.00
Yes	42	2.00 (0.99–4.06)	1.60 (0.76–3.35)	41	1.78 (0.99–3.21)	1.61 (0.87–3.00)	34	1.42 (0.75–2.67)	1.04 (0.44–2.43)
2001 (aged 18)		*p* = 0.08	*p* = 0.57		*p* = 0.19	*p* = 0.54		*p* = 0.630	*p* = 0.855
No	372	1.00	1.00	335	1.00	1.00	273	1.00	1.00
Yes	255	1.46 (0.95–2.24)	1.14 (0.74–1.75)	237	1.23 (0.90–1.67)	1.11 (0.80–1.53)	193	0.98 (0.65–1.50)	1.04 (0.63–1.47)

Maternal depression/anxiety	19 years (2001)			23–24 years (2004–2005)			30 years (2012)		
	*N*	Crude PR	Adjusted PR^a^	*N*	Crude PR	Adjusted PR^a^	*N*	Crude PR	Adjusted PR^a^
Females									
1986 (aged 4)		*p* = 0.64	*p* = 0.88		*p* = 0.03	*p* = 0.09		*p* = 0.278	*p* = 0.448
No	42	1.00	1.00	99	1.00	1.00	76	1.00	1.00
Yes	23	1.17 (0.60–2.30)	1.05 (0.54–2.04)	44	1.75 (1.05–2.92)	1.52 (0.94–2.45)	35	1.42 (0.75–2.67)	1.28 (0.67 to 2.45)
2001 (aged 19)		*p* < 0.001	*p* < 0.001		*p* < 0.001	*p* < 0.001		*p* < 0.001	*p* = 0.001
No	639	1.00	1.00	597	1.00	1.00	524	1.00	1.00
Yes	255	1.84 (1.53–2.23)	1.67 (1.36–2.04)	241	1.58 (1.30–1.92)	1.52 (1.23–1.89)	204	1.82 (1.37–2.40)	1.65 (1.20–2.13)

a Adjusted for family income, maternal schooling and household wealth index.

The prevalence of D&A was higher among male subjects whose mother reported D&A in childhood than if she reported an episode in adolescence, but all confidence intervals included the reference (see Table 3). Therefore, overall there was no convincing evidence for associations in male offspring.

### Maternal D&A in late adolescence and persistence/trajectories of D&A in female offspring during their transition to adulthood

**Fig. 2.**
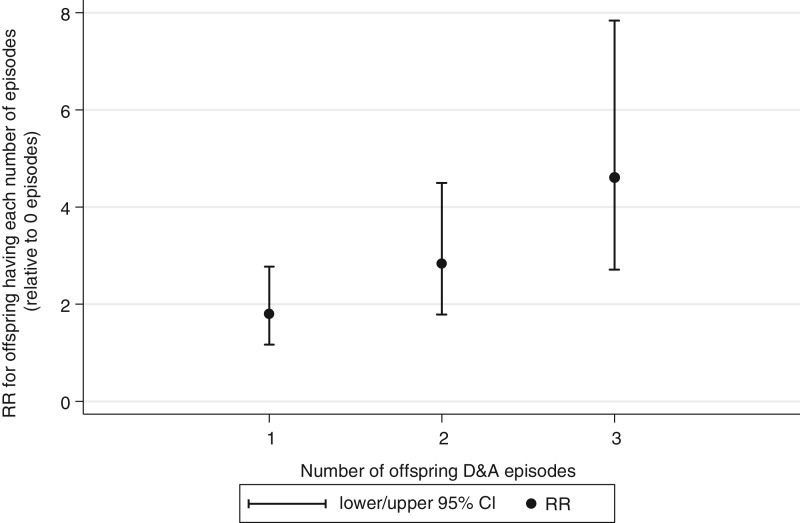
Offspring risk of having increasing depression and anxiety (D&A) episodes according to maternal D&A.

Fig. 2 demonstrates that maternal disorder in adolescence was associated with persistence of episodes in female offspring. Ordinal logistic regression analyses revealed that daughters whose mothers reported a disorder were more likely to show an increasing number of episodes than daughters of symptom free mothers [odds ratio (OR) 2.7, 95% confidence interval (CI) 2.0–3.7] and were 4.6 times (95% CI 2.71–7.84) as likely to report D&A at all three time-points between 19 and 30. There was no association in male offspring (OR 1.3 95% CI 0.9–1.9).

There was also evidence that maternal symptoms were associated with the intercept and slope of their daughter's symptoms from ages 19 to 30. On average females showed a SRQ-20 score of 4.5 (95% CI 4.1–4.9) at the first assessment (i.e. the intercept). On average females’ SRQ-20 scores rose by 0.48 points (95% CI 0.31–0.65) at each wave (i.e. the slope). Daughters of depressed mothers, started 2.69 (95% CI 1.93–3.45) points higher and rose on average 0.36 points (95% CI 0.03–0.67) more, at each wave than girls of non-depressed mothers.

On average boys have a SRQ-20 score of 1.43 (95% CI 1.03–1.83) at the first assessment (i.e. the intercept), on average the SRQ-20 scores rose by 1.0 point (95% CI 0.82–1.18) at each wave (i.e. the slope). Boys of depressed mothers, start 0.36 (95% CI −0.26 to 0.97) points higher and rise on average 0.02 points (95% CI −0.26 to 0.30) more at each wave than boys of non-depressed mothers.

### Specific constellations of maternal D&A and female offspring D&A at age 30

As shown in Supplementary Table S1, there was evidence that the only symptom constellation in mothers to be independently associated with female offspring risk was maternal ‘depressed thoughts’; PR of disorder according to maternal depressed thoughts was 1.25 (95% CI 1.0–1.57, *p* = 0.047), adjusting for all other maternal symptoms. Given that there was no main association between maternal and male offspring D&A this analysis was only conducted for females.

## Discussion

The current results represent a detailed investigation of the relationship between maternal and offspring D&A symptoms across offspring's transition to adulthood. As far as we are aware, this is the first study to address this issue from a low- or middle-income country and to investigate the impact of maternal disorders on the persistence of offspring symptoms.

Our results provide some evidence that maternal D&A measured in late adolescence was consistently associated with offspring D&A across the transition to adulthood. However, these associations appeared to be limited to female offspring. In addition there was evidence that maternal disorder was strongly associated with persistent D&A in daughters across all time-points. Finally there was evidence that a particular constellation of maternal symptoms (depressed thoughts) was independently associated with both daughters’ symptoms at 30 and the persistence of D&A symptoms across all follow-ups. Our results were inconclusive regarding the association between maternal depression at 4 years and later offspring depression. There was no clear evidence that D&A in mothers when their children were aged 4 years was associated with offspring D&A at later time-points because associations were inconsistent and confidence intervals were wide. However, some of the PRs were of similar magnitude to associations found between maternal D&A at age 19 and offspring depression (particularly in boys). Therefore, given the smaller sample at this age, caution should be taken in interpreting these findings as evidence that maternal depression at age 4 is not important for offspring depression. There was, however, a clearer female vulnerability following exposure to maternal D&A in adolescence, where there was statistical evidence for an interaction between maternal symptoms and offspring gender. Thus, although not conclusive, this raises the possibility of gender differentials in susceptibility to maternal emotional problems, which may vary with age of the offspring at the time of exposure (see below).

### Mechanisms and relation to previous research

In contrast to the British birth cohort study which found no effect modification by gender; in the current study there was clear evidence that the associations between maternal mental health in adolescence and later offspring mental health were stronger for daughters, both concurrently and well into adulthood. Caution should be exercised when looking at results split by gender due to the relatively reduced power. That said, in this study we did find statistical evidence for a gender interaction which increases our confidence in the finding. This female vulnerability is consistent with evidence from meta-analytical reviews of the association between maternal depression and internalizing disorders in childhood (Goodman *et al.* 2011).

As described in the Introduction, gender differences in the incidence of D&A emerge during adolescence with adolescent females experiencing a marked rise in emotional symptoms. One explanation for this difference is that females cope less well with stressors in adolescence (Goodman *et al.* 2011). These stressors may be particularly apparent in middle-income countries, with previous evidence reporting that females in Chile are more vulnerable to D&A than UK females and that this difference in gender susceptibility is associated with lower social support and resources for women (Rojas *et al.* 2005). Indeed, our earlier analyses show inverse associations between socioeconomic position and mental health scores (Anselmi *et al.* 2008). The mechanisms behind this effect seem to relate to the subject's ability to cope with stressful life events (WHO, 2010); including financial insecurity, limited work opportunities, gender discrimination and lack of family and social support (Patel *et al.* 1999; Ludemir & Melo Filho, 2002).

Thus, one explanation for our female specific findings is that during this difficult transition, girls are particularly dependent on their mothers for support. Where this support is compromised by maternal mental health problems, adolescent emotional symptoms may persist and develop into longer term disorders. For example, facilitated by attentive and supportive mothering, teenage girls may develop coping strategies for changing emotions. Maternal mental health disorders, however, compromise a mother's ability to provide such support and depressed mothers are more critical of their adolescents, which, is in turn associated with adolescent D&A (Nelemans *et al.* 2013).

In addition, during the transition from childhood to adulthood, females in particular, may be more likely to model their mother's behaviour (consciously or unconsciously). Adolescents of depressed mothers may, therefore, model their mother's ‘depressed’ ways of thinking and interpreting new female social roles, including gender discrimination, which they may experience at this time. Gender-specific socialization mechanisms may also explain this female vulnerability (Goodman *et al.* 2011). Mothers may feel it is more appropriate or acceptable to express negative emotions with their daughters than their sons. Mothers and daughters may also spend more time together due to more shared interests/roles. The lack of gender differences in the British cohort study (Schepman *et al*. 2011), raises the possibility that any such socialization mechanisms could be culturally specific.

There may also be methodological differences which could also account for observed differences. For example, in the current study we defined depression at different thresholds for males and females. A lower threshold for males is required given that males tend to underreport symptoms on screening tools. Therefore, if the same threshold was taken for males and females it would result in a substantially larger prevalence in females than in males and many false negatives in males. This would result in greater power to detect associations in female compared to male offspring. Taking the lower threshold for males resulted in similar prevalence of depression in males and females by age 30, indicating that by taking this approach our sex difference analyses were less influenced by relatively greater power to detect femal- specific associations.

Shared genetic risk factors are likely to explain part of the inter-generational association both through direct inheritance and correlations (both passive and evocative) between genetic and environment factors. Therefore, it remains possible, that the observed gender differences also reflect female-specific genetic vulnerabilities passed from mother to daughter and which may be activated during adolescence following the emergence of female hormones. However, large genome-wide association studies have provided no evidence for sex-linked genes for depression and there is no evidence that depression is more heritable in girls than boys (Thapar *et al.* 2012).

In addition, epigenetic change (modification of gene expression, such as through methylation, without changing the genetic sequence) is a mechanism that is proposed to explain the long-lasting effects of early life experiences on biological and behavioural phenotypes. Maternal mental disorders may alter maternal caregiving, which animal studies demonstrate lead to epigenetic changes in offspring. Thus epigenetic pathways may mediate the association between maternal disorder and child outcome, although this has not been formally tested (see Stein *et al.* 2014).

Interestingly, depressed thoughts in the mother were the only symptoms to show an independent association with risk of later mental health disorders and persistence of symptoms in daughters. Furthermore, maternal depressed thoughts were associated with a broad range of symptoms in daughters including, but not limited to, the same depressed cognitions. This explorative finding could provide further support for the role of parenting. Depressed thoughts and associated rumination, distraction and pessimistic interpretations of the family's life may compromise a mother's ability to attend to and provide positive emotional support and guidance. Further work is needed to replicate this finding and fully understand why these maternal symptoms may be particularly important. However, these findings suggest that in order to minimize the risk to the offspring ‘depressed thoughts’ specifically, may need to be targeted in intervention strategies and highlight the importance of considering specific maternal symptoms.

### Strengths and limitations

Strengths include the longitudinal design spanning over 25 years, the use of the same instrument over time as well as for both mother and offspring; the fact that three quarters of the cohort subjects were traced to the age of 23–24 years and two thirds to the age of 30 years, with no substantial socioeconomic differences between the sample lost to follow-up and those remaining in the study (Victora & Barros, 2006) and finally the availability of confounding variables.

Limitations include the fact that sample size of mothers with a SRQ-20 measure at age 4 was relatively small which affected the statistical power of the study to investigate the influence of maternal D&A in childhood on later outcomes. With 347 participants the study was powered to detect a correlation of *r* = 0.15, which is a moderate/weak correlation and smaller than the correlations observed at later timings; however, smaller correlations will not have been detected. This was not an issue when investigating the impact of maternal D&A in late adolescence due to a much larger sample at this point.

The SRQ-20 is a screening instrument, and does not provide clinically verified individual diagnoses. However, it has high sensitivity and specificity against clinical diagnoses (Mari & Williams, 1986). Males and females were assessed 1 year apart at the 18/19 time-point and within different contexts; however, this was not the case for the later measurements on which the main conclusions were based. Other psychiatric symptoms (behavioural, psychotic or personality) were not assessed. Given the co-morbidity of mental health disorders these could potentially be contributing to the observed associations. Although not conclusive given the exploratory nature of the symptom by symptom analysis, the findings that depressed cognitions seemed to be particularly important in the inter-generational associations; however, points towards a more depression-specific effect. Future studies should investigate the influence of co-morbid disorders in mother and offspring.

The role of maternal depression between the ages of 5 and 18 is also unclear, therefore we cannot be certain that maternal depression during adolescence specifically was important, the effects could have been driven by maternal depression in later childhood. Future studies with frequent measures of maternal depression are needed to disentangle the role of timings of maternal depression more thoroughly.

Finally fathers of the cohort were not assessed, which limits conclusions about the specificity of the influence of mothers.

## Conclusion

In conclusion, the inter-generational association between maternal and offspring D&A persists well into adulthood, highlighting the long-term importance of maternal D&A. From adolescence into adulthood, however, the inter-generational risk appeared to be limited to daughters. For daughters, maternal D&A was strongly associated with persistence of D&A episodes, which previous studies suggest are particularly important to future disorders. There is evidence for treatments which can reduce maternal depression (Howard *et al.* 2014); however, it should not be assumed that treatment of the maternal disorder alone reduces risk in the child (Murray *et al.* 2010; Stein *et al.* 2014). That said, there is promising evidence that targeting difficulties in the parent child relationship can reduce the impact of the mothers’ disorder on her child. Therefore it is important to consider modifying the maternal disorder itself but also modifiable pathways of risk such as parenting.

## Supplementary material

For supplementary material accompanying this paper visit http://dx.doi.org/10.1017/S0033291715001956. click here to view supplementary material
