# Supplementary material for: Maternal depression and anxiety predicts the pattern of offspring symptoms during their transition to adulthood
Source: Psychol Med. 2015 Oct 12;46(2):415–24. doi: 10.1017/S0033291715001956 (PMC4697191; doi:10.1017/S0033291715001956)
Supplement: Supplementary file 1 [file S0033291715001956sup001.docx]

**Supplementary Table S1. Prevalence ratio of DEPRESSION AND ANXIETY and each DEPRESSION AND ANXIETY symptom for female 30 year olds according to specific maternal symptoms in late adolescence, in a combined mutually adjusted model, thus estimates represent ‘independent’ associations.**

| **Mother's symptoms in 2001** |  | **Offspring symptoms at age 30**  **Any DEPRESSION AND ANXIETY** | **Depressed mood** | **Anxiety** | **Depressed thoughts** | **Poor concentration** | **Somatic** | **Sleep/Fatigue** |
| --- | --- | --- | --- | --- | --- | --- | --- | --- |
| **Depressed mood** |  | 0.95 (0.80; 1.12) p=0.529 | 1.02 (0.70;1.48) 0.934 | 0.95 (0.68 ;1.34) p=0.780 | 1.14 (0.71 ; 1.82) p=0.571 | 1.06 (0.75;1.48) p=0.754 | 1.00 (0.75;1.27) p=0.720 | 0.99 (0.73; 1.34) p=0.943 |
| **Anxiety** |  | 1.0 (0.96 ; 1.31) p=0.962 | 1.06 (0.734; 1.53) 0.755 | 1.06 (0.77; 1.46) p=0.722 | 0.73 (0.45 to 1.16) p=0.185 | 0.96 (0.70 ;1.32) p=0.824 | 1.12 (0.84 to 1.48) p=0.424 | 1.05 (0.79 ; 1.40) p=0.726) |
| **Depressed thoughts** |  | 1.25 (1.0 ;1.57) p=0.047 | 1.66 (1.11 : 2.49) p=0.015 | 1.54 (1.07 ;2.23) p=0.021 | 1.99 (1.20 : 3.30) p=0.007 | 1.23 (0.83 ;1.81) p=0.303 | 1.16 (0.82;1.66) p=0.387 | 1.64 (1.17 ; 2.30) p=0.004 |
| **Poor concentration** |  | 1.12 (0.96;1.31) p=0.164) | 1.08 (0.734; 1.53) p=0.755 | 1.04 (0.74;1.45) p=0.838 | 1.25 (0.78 to 1.98) p=0.354 | 1.02 (0.73;1.43) p=0.889 | 0.92 (0.69;1.25) p=0.628 | 0.91 (0.67 ; 1.24) p=0.561 |
| **Somatic** |  | 1.04 (0.91; 1.18) p=0.558 | 0.82 (0.56 ;1.18) p=0.278 | 1.15 (0.84; 1.58) p=0.391 | 1.27 (0.81 to 1.98) p=0.298) | 0.83 (0.59;1.15) p=0.264 | 0.95 (0.71 ;1.27) p=0.720 | 1.04 (0.78 ; 1.40) p=0.783 |
| **Sleep/Fatigue** |  | 1.15 (0.91 ; 1.35) p=0.085 | 1.87 (1.25; 2.8) p=0.002 | 1.29 (0.89 ; 1.86) p=0.173 | 1.15 (0.68 to 1.95) p=0.590 | 1.65 (1.15;2.37) p=0.006) | 1.23 (0.89 ;1.71) p=0.208 | 1.34 (0.96 ;1.87) p=0.084) |

**Depressed Mood** = (Do you feel unhappy? Have you lost interest in things? Do you cry more than usual? Do you enjoy your daily activities? )

**Anxiety** = ( Are you easily frightened ? Do you feel nervous, tense or worried ? Do your hands tremor?)

**Depressed thoughts/ Hopelessness** = ( Do you feel a worthless person? Has the thought of ending your life been in your mind? Are you unable to play a useful part in life?)

**Poor concentration** = (Do you have trouble thinking clearly? Do you find it difficult to make decisions ? Is your daily work suffering?)

**Somatic** = (Do you often have a headache? Is your appetite poor?, Is your digestion poor? Do you have uncomfortable feelings in your stomach?)

**Sleep/Fatigue =** (Do you sleep badly? Do you feel tired all the time? Are you easily tired?)

***Note:*** Subjects were categorized as experiencing the symptom if they endorsed 2 or more symptom specific items
